# Parents’ Experiences Using Digital Health Technologies in Paediatric Overweight and Obesity Support: An Integrative Review

**DOI:** 10.3390/ijerph20010410

**Published:** 2022-12-27

**Authors:** Torbjørg Goa Fidjeland, Kirsten Gudbjørg Øen

**Affiliations:** Department of Public Health, Faculty of Health Sciences, University of Stavanger, 4036 Stavanger, Norway

**Keywords:** digital health, technology, paediatric obesity, pediatric obesity, weight management, parents, experience, user perspective, parental support

## Abstract

This study aimed to explore parents’ perspectives regarding the use of digital health technologies in paediatric overweight and obesity weight support. A systematic search in ‘Academic Search Premier’, ‘British Nursing Index’, ‘Cumulative Index to Nursing and Allied Health’, and ‘Health Research Premium Collection’ was conducted in November 2021. Inclusion criteria were English or Nordic peer-reviewed articles published after 2010, overweight and obese children aged 2–10, weight support using digital health technologies, and parental experiences examined. An integrative review was conducted using Joanna Briggs Institute quality appraisal checklists and a constant comparison analysis method. In total, 14 articles were analysed that included SMS, telephone, apps, websites, and social media as the main technology groups. A limitation of this review was the heterogenicity of the studies. The results indicate that parents, mostly mothers, had a positive experience, finding technologies helpful and easy to use, and expressed optimism toward future use. An option for interacting with others and the possibility of personalising support were enjoyed or requested. In conclusion, digital health technologies can be a suitable tool to empower the parents of children with overweight and obesity concerns, whose perspective should be considered during the design and support phases.

## 1. Introduction

Globally, about 18% of children and adolescents were overweight or obese in 2016 [1]. The prevalence of overweight and obesity issues among Norwegian primary school children is of concern, as 17% of children aged 6–11 years are overweight or obese [2]. The high incidence of overweight and obesity problems among children shows that this is a major challenge. Internationally, various definitions and calculation methods for overweight and obesity measures are used [3]. In Norway, an age- and gender-adjusted body mass index (iso-BMI) is used to assess overweight and obesity issues among children, where BMI = $\frac{\mathrm{kilogram}\;\left(\mathrm{kg}\right)}{\mathrm{height}\;\mathrm{in}\;\mathrm{centimetres}\;{\left(\mathrm{cm}\right)}^{2}}$. For children aged 2–18, iso-BMI ≥ 25 = overweight, and iso-BMI ≥ 30 = obesity [4].

Obesity is regarded as a complex condition. Discrepancies in the energy balance are claimed to be the most common cause, but biological, developmental, behavioural, genetic, and environmental factors also influence body weight [5]. Being overweight or obese can have several serious negative consequences for health, both of a mental and physical nature [5]. The development of overweight and obesity occurs gradually. When working with overweight and obese children, the aim is to stabilise weight gain while continuing height growth, thus reducing the degree to which they are overweight [6]. For children, parents are the most important influence in promoting a healthy lifestyle [7,8,9], making them crucial participants in weight and lifestyle support. 

Digital health can refer to ‘a wide range of technologies directed to delivering healthcare, providing information to lay people and helping them share their experiences of health and illness, training and educating healthcare professionals, helping people with chronic illnesses to engage self-care and encouraging others to engage in activities to promote their health and wellbeing and avoid illness’ [10] (p. 1). Currently available technologies include email, apps, SMS and audiovisual communication between patients and healthcare providers, and health-related web pages, online discussion forums, and social media [10] (pp. 3–4). Digital health holds great potential, i.e., economically, through remote treatment, reaching a high number of persons with high-quality and low-cost services, and allowing patients to gain more autonomy. However, there are also negative aspects, such as data security and systems being created more for the ‘system’ than the patient, alienating the patient in the process and risking further marginalisation [11]. Using digital health care may also demand consideration of the patient’s safety, welfare, and consent issues. 

Digital health technologies used in the healthcare system for paediatric weight support are explored in terms of their ability to meet the needs of families with children with overweight and obesity. Internet-based interventions and telemedicine have, among other topics, been discussed as strategies to meet families’ needs and logistical difficulties and to reduce attrition, a common challenge in paediatric health management [12]. Feedback from parents suggests that using digital health technologies is an acceptable approach to promoting a healthy lifestyle [13]. The support should be easy to use and low or no cost [14]. However, previous studies report mixed results on BMI and lifestyle changes when using digital health technologies [15,16,17]. 

Culturally competent or individualised approaches are needed to reduce attrition in paediatric weight management [18]. The user perspective is vital to ensure that interventions meet the needs of users [14]. Empowerment can be defined as ‘the process by which people gain control over the factors and decisions that shape their lives’ [19]. Empowering parents in the intervention design and implementation processes for paediatric overweight and obesity prevention and management has shown promising results, but often parents do not participate or contribute to the development of weight interventions [8]. In one review on mobile-based paediatric obesity prevention, only 33.3% of the studies examined the participants’ experiences of the intervention [20]. 

User experiences can influence digital healthcare innovations and help adjust innovations to meet the need of the users better. Consequently, they have been considered in several digital health innovations targeting paediatric obesity prevention and management [21,22,23]. Despite the importance of including the user perspective, it is difficult to find studies examining user experiences among parents of children who are already overweight or obese. 

This review is one part of the ongoing action-oriented project ‘Healthy Future 2’ (HF2), initiated by the University of Stavanger and building upon the project ‘Healthy future’ [24]. HF2 is designed to contribute to preventing more children from developing obesity through adequate healthcare support to parents and families. The overall aim of HF2 is to develop, implement, and evaluate a Healthy Future Tool and a digital, knowledge-based platform for healthcare professionals and parents of overweight children aged 2–7. The project is designed to engage both parents and practitioners in all stages of the development of the intervention [25]. Research shows that early intervention is important in the event of incipient obesity. Unfortunately, this kind of care is underdeveloped in the Norwegian healthcare system [25]. As key professionals in child health clinics, public health nurses (PHN) are often the first healthcare professionals overweight and obese children meet. They play a vital role in all stages of paediatric overweight and obesity prevention and management [4]. 

This study aimed to explore parents’ perspectives regarding using digital health technologies in paediatric overweight and obesity weight support. The research questions were: (1) What are parents’ experiences using digital healthcare in paediatric weight support? (2) Do parents have any wishes for future weight support programmes based on these experiences? The focus was on technology use. The goal was to explore how parents in previous studies had experienced digital paediatric overweight and obesity healthcare in terms of sharing their perspectives, thus starting to include user perspectives in the development of new and sought-after healthcare for both PHNs and others working with paediatric obesity. 

## 2. Materials and Methods

The literature search revealed different designs used in studies exploring parental experiences with digital health in paediatric weight management. Therefore, an integrative review, using Whittemore and Knafl’s (2005) description of the methodology, was used. This methodology is the only approach that can accommodate studies of several different designs, both experimental and non-experimental, unlike, for example, a systematic review [26] The integrative review consists of 5 stages: problem identification stage, literature search stage, data evaluation stage with quality appraisal, data analysis stage, and presentation stage [26].

Based on the aim and research questions of the study and experience from the initial searches, the following inclusion criteria were set: (1) Some of the children included had to be between 2–10 years old, although some of the participants might be older or younger; (2) Children must already be overweight or obese, based on the definition in the country of origin of the study; (3) The support should contain digital health elements, but the exclusive use of digital technology was not required; (4) Experiences of parents, caregivers, or other family members were assessed, and the experiences were distinguished from those of the clinicians or children. For simplicity, parents (both mothers, fathers, stepparents, co-parents, foster parents, and adoptive parents), caregivers, and other family members are hereafter referred to as parents; (5) The user experience had to explore more than just whether parents liked the intervention; (6) Peer-reviewed primary studies published after 2010, in the Nordic or English language, these three limitations filtered automatically in the databases. 

The literature search strategy was designed with the help of a librarian, using a PEO (Population/Exposure/Outcome) search strategy [27] (Table A1). In the process of building the search, both test searches and a hand search of ‘Telemedicine journal and e-health’ were conducted to find relevant articles and keywords. The main search was conducted in November 2021 in the following databases: ‘Academic Search Premier’, ‘British Nursing Index’, ‘Cumulative Index to Nursing and Allied Health’, and ‘Health Research Premium Collection’ (including MEDLINE). The search was transferred to Zotero, where duplicates were removed. The remaining articles were then assessed using rayyan.ai. Manual searches in the reference lists of included studies and reviews on the topic were also conducted to have multiple search strategies, as recommended in the relevant methodology [26].

Because of the methodological diversity in the primary studies, the selected studies were quality appraised using multiple Joanna Briggs Institute (JBI) critical appraisal checklists. How to appraise studies from diverse designs is discussed. Using a multiple quality evaluations checklist renders quality appraisals more complex, but also more suited for the different research designs used [26]. The approach toward low-quality studies in an integrative review, whether to leave them in for diversity or exclude them because they may lead to inaccurate conclusions, was deferred [28]. After a discussion between researchers, we chose to eliminate studies of poor quality. 

All data regarding parents’ experiences were extracted for analysis, and we also collected bibliographic information, sample characteristics, study methodology, intervention, digital use, and the main finding(s). The analysis was performed using a constant comparison method, an analysis method argued to be compatible for varied data from diverse methodologies. The analysis method follows four phases. The first phase consists of data reduction. The next phase is data display, then data comparison in the third phase, before conclusions drawing and verification of the findings in the final phase [26]. Microsoft Excel was used in the data reduction and data display steps. Both Microsoft Excel and Microsoft Word were used in the data comparison and conclusion-drawing steps. Findings from both the Microsoft Excel matrix and Microsoft Word analysis were verified with the primary studies in the last step. Both an overall analysis of all included studies and a subgroup analysis of each main technology group were conducted, following the same approach. 

The review included already published studies, thus no ethical approval was needed in advance. All included studies had been given ethical approval in advance.

## 3. Results

### 3.1. Selection

**Figure 1:**
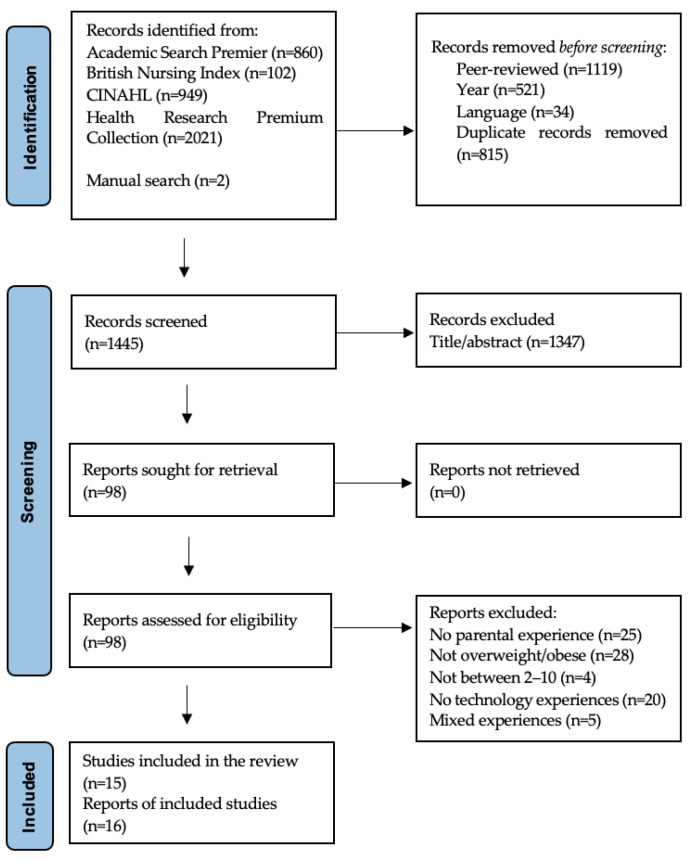
PRISMA flowchart [29].

A total of 3932 records were identified in the initial search, and 2491 records were removed automatically by filters. Two records were later identified from the reference lists of the included studies and reviews on the subject and added to the flow chart. A final number of 98 reports were assessed for eligibility, leaving 16 reports from 15 unique studies to be appraised for quality. A flowchart of the study identification process is shown in Figure 1. 

See Table A1 for the full search strategy.

The reviewed articles included eight randomised controlled trials (RCTs) from seven different studies [30,31,32,33,34,35,36,37], three qualitative studies [38,39,40], one mixed method approach [41], one experimental design [42], and three quasi-experimental studies [43,44,45] in the final sample. Two of the articles [31,36] were part of the same study. One study did not meet the overweight/obesity-only criteria, but included children with abnormal weight gain (ICD-9 code 283.1) who were referred to weight management clinics [42]. After discussion, the researchers decided to include this report in the review because these children are in the target group for getting weight support from healthcare professionals (HCPs) [46]. 

### 3.2. Quality Appraisal

The studies were quality appraised using Joanna Briggs Institute (JBI) critical appraisal checklists [47,48,49]. The mixed methods study [41] was appraised using both the qualitative research checklist [48] and the quasi-experimental studies checklist [49]. 

All studies, except two, obtained an overall score of over 50% on their respective checklists. The two studies with the lowest score had some methodological challenges and were excluded. 

See Table A2 for the full quality appraisal. 

### 3.3. Study Characteristics

After the quality appraisal, 14 articles from 13 different studies were included in this review. Ten articles (nine different studies) were from the United States, all of which were published between 2011–2019. The articles from other countries were all published between 2019–2021. The aim of all the studies included some form of feasibility, experience, fidelity, acceptability, or satisfaction. Most studies also aimed to examine the effect of the intervention on BMI and/or designing a healthier lifestyle [30,33,34,35,36,37,43,45]. All quantitative studies had some form of a survey examining the experience, and four studies also included quantitative feedback [32,34,43,45]. The qualitative studies gathered the information through focus groups [38], individual interviews [40], or both [39]. 

There were five main groups of technology used: eight used SMS/email (all eight studies had an SMS option, and some studies also let the participants choose between SMS and email, using SMS or email in the same way) [30,31,32,34,35,36,39,43]; two used social media [32,38]; seven used video calls and phone calls [31,32,33,36,37,42,45]; five used web-based systems [31,32,35,36,40]; and two used apps [34,45]. The number of digital components used in the different studies varied from 1 to 4, with a slow rise in the number of digital components in more recent studies.

All articles, except four, had some form of limitations regarding underlying illness for the children who participated. Three studies [35,42,43] did not specify, and one study [38] had a medical diagnosis as an inclusion criterion. The study settings were diverse, with both rural [33,42] and low-income neighbourhoods [30], as well as studies with diverse socioeconomic settings. Two of the studies also included other caregivers (grandparents, guardians, and aunts) as adult participants in addition to parents [38,42], while the rest considered parents exclusively, mostly mothers. The list of included studies, characteristics, main findings, description of the interventions, and the parental digital technology used, if reported, are shown in Table 1 and Table 2. 

### 3.4. Findings

The analysis indicates that most parents had an overall positive experience with using digital health technology in paediatric overweight and obesity support; they also had some suggestions for future use. A total of four themes emerged: Positive experience (1); Technology easy to use (2); Importance of interaction (3); and Essential personalisation (4). Table A3 provides a keyword table of their experiences to illustrate the analysis. 

#### 3.4.1. Theme 1: Positive Experience 

The overall satisfaction rate was high across different studies. In one study, 78% of the parents in the intervention group using the digital component agreed that their expectations of the obesity treatment were met, while only 22% of the control group felt the same (*p* = 0.057) [34]. All included studies described one or more of the following factors: satisfaction, helpfulness, ease of understanding, convenience, enjoyment, usefulness, suitability, timing, appropriate frequency, or if they would recommend the programme to a friend, marking this as the most examined type of experience. Most parents reported positive feedback on these aspects, with many studies reporting more than 85% of parents being positive [30,31,32,33,34,35,42,43]. The quote, ‘The text messages were the best part of the whole programme. They were a helpful and handy reminder when you have started to slip and forget goals. They had helpful hints for conversations, and I really liked them’ [32], illustrated how the participants found texts helpful. In one of the early studies, parents suggested an option to self-regulate the frequency of messaging [39], a feature made possible in one of the later studies [43]. 

Answers to the question of whether the participants would like to continue to use the programme were more split: Facebook and SMS intervention had the lowest score (46–62%), while telehealth, app, and web-based programmes all had a score over 90% [30,32,37,42,45].

Parents liked to use digital technology for weight support because they saved time on travel and money; they found it practical to be able to participate when convenient, including participating from home [37,38,39,40].

#### 3.4.2. Theme 2: Technology Easy to Use 

The technology was easy to use for most parents [31,32,37,38,39,40]. Those experiencing challenges reported issues with remaining connected during the visit, login difficulties, difficulty being heard or seen, having problems downloading the software, space limits, costs, or the technology being too complex to use without support. Not having a compatible device or different preferences and confidence in the technology were other reasons [31,32,39]. One intervention chose to adjust the procedure because parents initially did not think they should respond to the SMS, resulting in a low response rate [43].

Based on the findings, it was impossible to draw a conclusion regarding what type of technology parents preferred. Even in some studies, parents expressed contradictory wishes regarding preferences [32,37,39]. 

#### 3.4.3. Theme 3: Importance of Interaction 

Some form of interaction between participants or clinicians, either by phone, SMS, or another method, was considered positive or suggested to incorporate if not already there [31,32,34,39,40]. Getting children involved was considered positive or, when it was not included, was suggested for incorporation. Parents also wanted increased participation when they found themselves minimally involved [32,38,39,40], an example being SMSs targeting children [32,39]. Interaction with peers was also regarded as positive on paper, although the experience was more mixed [32,38,39,40]. 

#### 3.4.4. Theme 4: Essential Personalisation

Parents in different studies wanted personalised weight support. Some parents found all or part of the intervention personalisable in that it gave specific suggestions [30,32,43], while others wanted more personalisation and specific suggestions [32,39]. For example, ‘“Your kid should exercise”. Really? I know that. Or, like, “Drink water”. Come on; we know that. Give me something interesting, like there was a new study out, and your kids should eat more of this’ [39]. 

**Figure 2:**
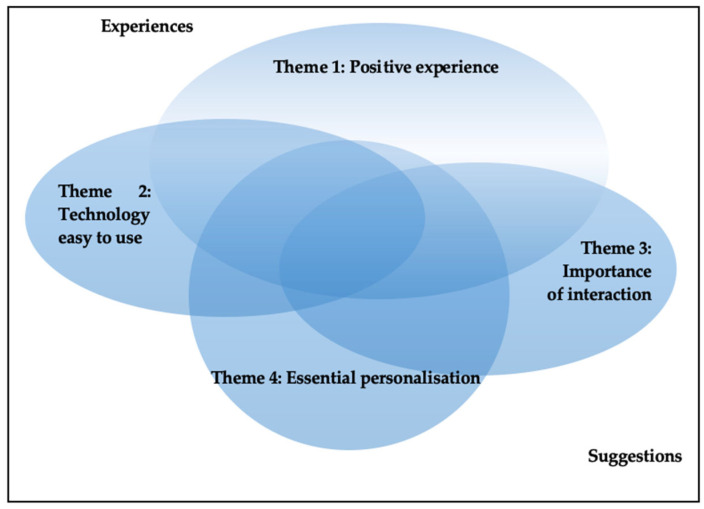
Illustration of the findings represented by the four themes.

Different approaches to personalisation were mentioned. One way was to customise the information to support behavioural change, which included using the participant’s name or adjusting the information to the child’s age, gender, neighbourhood, health conditions, preferred goals, and current knowledge [39]. Parents expressed a different need for the amount of desired information, which information they needed, their available time, and how long they felt the support should last [32,40]. Taking advantage of the possibility technology holds can also be seen as a method of personalising support. Factors such as around-the-clock access; asynchronous support; individual settings for frequency, timing, amount, and level; or interacting with others have been discussed in the other themes, but can also be seen as a way to personalise support, combining the themes as illustrated in Figure 2.

#### 3.4.5. Different Groups of Digital Health Technologies

Following the observation that the findings in the overall analysis were reflected in the different technology groups, subgroup analyses were conducted.

Most themes were discussed in all subgroups, although the smaller number of included studies in each subgroup analysis made the results less robust. This finding was particularly applicable to the app and social media groups, consisting of only two studies each. Personalisation was not mentioned by a majority in the app group, although it was also mentioned here by a user who mentioned multiple choices as positive [45].

There were some differences in the sub-analysis compared to the overall analysis. The phone-, app-, and web-based groups were more positive towards future use than social media and SMS groups (see theme 1). In the social media group, parents in one study had a positive experience [38], while the other had a more negative experience [32], leaving it impossible to draw conclusions. 

## 4. Discussion

### 4.1. Findings

This study aimed to explore parents’ perspectives on the use of digital health technologies in paediatric overweight and obesity weight support. The overall positive experiences with digital health technology are mainly comparable with other studies from different areas in paediatric healthcare [50] and other areas of paediatric overweight and obesity prevention and support [51,52,53]. The positive aspects of personalisation and interaction are also supported in previous research in paediatric healthcare [54] and other fields of paediatric obesity healthcare, e.g., prevention [14,20,21]. Differences emerged regarding preferred technology, method, design, and features between genders and age groups [55], which support the need to adjust the support to the user. 

A tailored approach should include consideration of the family’s health literacy, because there is good evidence that low levels of health literacy are associated with obesity in children [56]. Use of the ‘teach-back’ method, by asking the patient to repeat in their own words what they have understood, could be a means to ensure their understanding [57].

Some previous research on digital healthcare in various fields of paediatrics has a stronger emphasis on barriers [50,54]. Families’ willingness to try technology may have changed since the COVID-19 pandemic. We reveal that most participants found the technology easy to use, and the focus on barriers was not dominant when parents’ experiences were explored. However, a minority noted perceived difficulties and not being comfortable with the technology as reasons not to use digital health technology [31]. 

The participants in this review all agreed to participate in research involving the use of digital health technology. One objection may then be that the sample is not representative of the entire population, as those who choose to participate tend to be more comfortable using technology than those who decline to participate. Nevertheless, the same population bias applies to other research as well; thus, it is difficult to determine the reason for the inequality. However, this potential population bias becomes important when using digital health technology across the entire population. Here, one should also consider that much of the findings on experience were obtained late in the interventions. At this point, many of the participants lost to follow-up were no longer participating in the study, making their voices lost in this review. There is a possibility that some of the participants who had negative experiences were in the lost-to-follow-up group; as a result, findings in this review may be more favourable than if all participants were included. 

A critique of digital health is that the technology can be created to suit the ‘system’, alienating the patient in the process. Incorporating the user in the innovation and empowering them throughout the whole process is proposed as a way of reducing this risk [11]. In addition to the innovation process, the findings also indicate that users want some influence on overweight support. Parents in the different studies experienced or wanted personalised weight support adapted to their needs [30,32,39,43]. Apart from the ‘live’ use of digital health technologies, like video calls or chats with HCPs, families have complete control over when and how much they use this service. Thus, making the service attractive and encouraging its use is essential. The findings indicate that allowing families to influence the support is important for promoting use.

Parents expressed different needs regarding content and their desired level of information and support [32,40]. In two of the web-based studies, parents accessed the device-friendly webpage with different devices based on their own preferences [32,35]. In one of the other studies, parents changed the timing and frequency of SMSs, and they also participated in selecting goals and the language used in the texts [43]. In these examples, parents influenced the finished product to suit their needs. Using the advantages of technology, with multiple choices in different areas, such as access to information about remaining active and cooking, can empower parents and grant them control over overweight support, customising it to meet their needs. The ability to choose the timing, amount, and frequency of information can further empower parents and reduce the barriers to participating in digital weight support. 

### 4.2. Implications, Future Research, and Limitations

The overall positive experience of parents using digital health technology in paediatric weight support can be used as an argument for why digital healthcare in paediatric overweight and obesity support is worth further exploration. It also bolsters the argument that creating a digital tool for this support, as intended via HF2, is worth doing.

However, the analysis could not draw conclusions about which tool to use. One challenge is the heterogeneity of the studies in the review. Others have also faced this challenge [14,20,55], making it an interesting topic for future research. The settings and socioeconomic backgrounds in the studies are diverse. An interesting research area is whether the same results will apply to different groups of participants or settings. 

Although we initially wanted to include all parents and caregivers of the children, it turned out that most of the respondents in the various studies in this review were mothers. The experiences of fathers and other caregivers are, thus, underrepresented in this review and may be an interesting topic for further research.

In this review, only two studies [32,37] used digital health exclusively. The others were either optional [31,36], both [30,34,35,38,40,43,45] or had some form of interaction with an on-site representative [33,39,42]. In other reviews, the combination of on-site and digital health support was also found [17]. Therefore, the findings in this review are mainly applicable to parents who receive both digital and on-site support, making it intriguing to find out whether parents in exclusively digital health interventions have the same experiences and desires. 

This review concentrated on parents’ experiences, leaving out other aspects of digital health. Issues such as legislation regarding the use of digital technology in healthcare, journaling, data security, and privacy have not been considered here. However, these topics must be addressed before any use of digital health technology. This review also does not analyse what content the support should contain, although some suggestions on how the content should be presented have been discussed. Implications for healthcare professionals and the healthcare system were not addressed, but must be respected in the development and implementation of new procedures. 

Drop-out rates in paediatric weight management are high. It is hard to find an estimate, as results vary, but a drop-out rate between 0–73% across different studies has been observed [58]. In this review, the drop-out rate was 0–35%. However, there is a considerable potential bias in the selection of studies here, as only studies that include parental experiences are included. Digital health care and retention rates can, thus, be an additional area for future research. 

### 4.3. Methodology

The heterogeneity of technology use and support in the included studies makes it relevant to question whether it is possible to compare the findings across the studies. This challenge was discussed among the researchers repeatedly throughout the process. Nevertheless, we chose to maintain the breadth of technology use and retain the broad purpose of the study to gain a better overview of different experiences. We also found that half of the studies included multiple components. Even though some of the studies analysed the different components separately, parental experiences are discussed in the full sample, which allows one to argue that a division of the components is complex. 

The analysis also reveals that parental experiences and future desires are quite similar across different types of technology. The search also revealed few studies on the topic, making a comprehensive approach appropriate. To account for the wide use of technology, we chose instead to reduce the age group included and exclude prevention, concentrating on weight support for those already experiencing overweight and obesity concerns. This choice between different combinations of limitations has emerged in other reviews of digital health and paediatric overweight and obesity concerns, which include limits regarding the type of technology, children’s age, methodology, or whether to include both prevention and management [15,17,59,60,61]. 

The novelty of the digital health research area made it necessary to conduct an exhaustive search because we found the use of terminology inconsistent. The inconsistent terminology and wide search make it possible that relevant studies have been overlooked. To prevent this, a librarian helped set up the search. The methodological literature recommends multiple search strategies to compensate for the lack of accuracy in a search due to inconsistent terminology and indexing [26,62]. By performing a hand search in one journal, as well as searching the reference lists of included studies and related reviews on the topic, we tried to get a more comprehensive search.

Because of the diversity in methodology in the relevant studies, we conducted an integrative review based on Whittemore and Knafls’s [26] updated description of the method. By following this procedure closely and including tables and references that clearly describe the interpretation of the findings, we sought to create transparency in the process. To ensure that important elements were addressed, we used the PRISMA 2020 item checklist [29], Table S1. Not all the items on this list have been addressed because of no relevance to this integrative review.

## 5. Conclusions

Using technologies to empower parents of children with overweight and obesity concerns can be a powerful tool in preventing and managing these issues. As in other services for this group, it is important to consult the user’s perspective and listen to them throughout the healthcare programme, including what kind of technological or face-to-face support they would prefer; offering tailored support is recommended. 

When planning to develop parental support approaches for families with overweight and obese children, parents should be invited early in the development process so their preferences can guide the service development. This approach might be a way to empower the patient and increase the health promotion processes so that parents can prevent further weight gain in their children. For further research, we recommend trials evaluating novel technologies with this population build in robust assessment of parental acceptability.

## Figures and Tables

**Table 1:** Included studies part 1, Demographics and findings.

Study, Country, Design	Children’s Age Criteria (Mean Age Included), Number of Participants, Number Lost, Percentage Lost	Main Findings
Armstrong et al. (2018) [30]. USA, RCT	5–12 (9.9), 101 dyads, 19 lost, 18.8%	No BMI difference, reduced attrition for a visit. SMS feasible.
Bala et al. (2019) [31]. USA, RCT	2–12 (8,0), 721 children, 57 lost, 7.9%	High satisfaction with technology components. Telehealth accessible and feasible approach.
Chai et al. (2021) [32]. Australia, RCT	4–11 (9), 46 dyads, 16 lost, 34.8%	Overall high satisfaction. Components were easy to use. Telehealth consultations led to good adherence. The programme improved eating habits according to parents. Would like to continue using/recommend to others.
Davis et al. (2016) [33]. USA, RCT	5–12 (9.14), 103 dyads, 8 lost, 7.76%	Both telemedicine and telephone appear feasible and acceptable methods with high satisfaction levels. Completion rate is high. No significant changes in BMI, physical activity, diet, or psychological effects in either group.
Johansson et al. (2020) [34]. Sweden, RCT	5–12 (8.4 and 9.8), 28 children, 3 lost, 10.7%	BMI reduction and attendance were better in the intervention group. Parents and clinicians found the experience positive and accessible.
Lee et al. (2019) [38]. China, Qualitative	8–16, 20 family caregivers	The mHealth intervention was well-received, and caregivers found it useful.
Militello et al. (2016) [43]. USA, Experimental	3–5 (54.47 months), 15 children, 13 parents, non-lost	Acceptable and feasible. The intervention improved parental knowledge, beliefs, and behaviours towards a healthier lifestyle.
Mulgrew et al. (2011) [42]. USA, Cross-sectional	Under 12 years (6.3 and 8.1), 25 surveys, 10 telemedicine surveys	No significant difference between most groups; slightly easier to understand explanations about children’s health in the face-to-face group. All would use telehealth again.
Sharifi et al. (2013) [39]. USA, Qualitative	6–12 (8.7), 31 parents	High acceptability. 2–3 SMS/week appropriate. Want relevant and personalised information. Links can be provided if necessary.
Sze et al. (2015) [35]. USA, Experimental	8–12 (11), 20 dyads, 0 lost	High adherence, easy to use, helpful and useful.
Taveras et al. (2017) [36]. USA, RCT	See Bala et al. (2019) [31]	Both groups improved family-centred outcomes for childhood obesity and improvements in child BMI and parental empowerment.
Thorén et al. (2021) [40]. Sweden, Qualitative	5–13, 14 parents	Parents found the programme helpful for the whole family to introduce a healthier lifestyle, predominantly positive experiences.
Tripicchio et al. (2017) [45]. USA, Experimental	2–18 (9.6), 64 children, 16 lost, 25%	Technology components were highly acceptable. Group 3 had significant BMI reductions, but the other groups did not.
Wright et al. (2013) [37]. USA, RCT	9–12 (10.3), 50 dyads, 7 lost, 14%	High interactive voice technology (IVR) users decreased BMI compared to low users. Of those who made calls, >75% agreed that the calls were useful, suitable, credible, and helpful.

**Table 2:** Included studies part 2, Intervention and use.

Study	Duration and Intervention	Parental Digital Use
Armstrong et al. (2018) [30].	12 weeks. Group 1: monthly health visit. SMS-reminders. Group 2: same as group 1 + daily SMSs (Monday–Friday). The first SMS of the week was a text-based dialogue.	Parents responded at least once to 80% of SMSs, and twice or more times to 30%.
Bala et al. (2019) [31].	1 year. 1st group: monthly SMS and email of neighbourhood resource guide. 2nd group: 6 individual sessions (in-person, video call, or phone), twice weekly SMSs, possibility to reply and receive automated age-specific feedback, GIS map of the neighbourhood and referrals to community resources. Choice between SMS and mail.	2nd group: 93% chose SMS. 99% responded to at least one SMS, and 61% responded to more than half. 28% used video calls at least once. Parents used video calls in 14–23% and phone calls in 69–86% of visits.
Chai et al. (2021) [32].	12 weeks. Group 1: 2 video consultations with dietitian, website and Facebook group posting summary with website links as prompts. Group 2: same as group 1 + SMS targeting both parents with a link to the website. Group 3: Waitlist, all components at week 12.	Telehealth: 78–96% attendance, 86% attended by mother and child, 82% in the afternoon. Devices used on the website: desktop (66%); mobile (27%); tablet (7%). Visits through links (64%).
Davis et al. (2016) [33].	8 months. Group-based intervention (8 weeks weekly, then monthly). Off-site leaders met parents via telemedicine or speakerphone; on-site school representatives met with the children. Meetings lasted approximately one hour.	Overall attendance rate: 89.40%.
Johansson, et al. (2020) [34].	Group 1: standard care. Group 2: standard care + daily weight, SMS communication between parents/clinician, activity monitor connected to a gamified app for children.	Weight frequent at the beginning, than sable at 2.4/week. Messages median (IQR) frequency of 4 (6), ranging from 0 to 13.
Lee, et al. (2019) [38].	Mixture of seminars and workshops, both physical and using mHealth (WhatsApp and Messenger). The research team uploaded the information to the mHealth tools.	
Militello et al. (2016) [43].	7 weeks. 4 parts: (a) face-to-face visits; (b) reminders (manual); (c) triggers (SMS); and (d) reinforcements (homework). Participants responded to SMSs twice weekly, receiving automated feedback. Parents also developed skill-trigger SMSs, where they select the skill, verbiage, and day(s) and time to receive SMSs.	The number of SMSs sent: 7–39, mean 22.31 (SD 9.47). 69% changed the frequency of tailored SMS (1–5 /week). 58.8% of SMSs sent in the afternoon. The initial response rate for conversational SMSs only 26%. After shortening and simplifying, the increased response rate is 80%.
Mulgrew et al. (2011) [42].	Cross-sectional survey assessing parent satisfaction: Group 1: telemedicine group seen at rural clinics, with a rural provider in the clinic and paediatrician and dietician present via video call; Group 2: regular on-site visits.	Telemedicine group: 70% answered the survey after the first visit. 30% had had 2–9 prior telemedicine consultations (Mean 1.6).
Sharifi et al. (2013) [39].	Two phases: (A) participants received SMS during focus groups to discuss the experience; (B) some participants reviewed a 3-week mock intervention, receiving 3 SMSs/weekly.	
Sze et al. (2015) [35].	4 weeks. Preintervention nutrition intervention for all. Group 1: personally tailored, device-friendly interactive website, where participants generated their own cues to listen to at least 2/daily + SMS/email prompts 2/daily, weekly sessions. Group 2: same + own audio recordings of episodic future thinking x2/daily.	Devices used: Adult group 1: desktop 21.3%, tablet 16.5%, mobile phone 62.2%; Adult group 2: desktop 34.2%, tablet 2.7%, mobile phone 63.1%.
Taveras et al. (2017) [36].	See Bala et al. (2019) [31].	
Thorén et al. (2021) [40].	4 weekly group sessions, followed by 12-week web-based programme targeting parents and children. Weekly modules. Device-friendly. Participants also received physical activity on prescription.	Most parents used the web-based weekly coaching modules. All families registered in the web-based programme.
Tripicchio et al. (2017) [45].	12 weeks. Group 1: weekly 2-h group sessions. Group 2: Same + tablets with fitness app with instructions for use. Group 3: Same as group 2 + individual video calls every other week.	81.3–100% used the app at least once. Group 3 used the app significantly more than group 2. Families received at least one call, and 44.5% received five sessions or more. Calls lasted, on average, 0.5–1 h.
Wright et al. (2013) [37].	12 weeks. Parents and children received telephone counselling intervention delivered by automated IVR. Participants called twice/week, option to complete both at once. IVR asked questions and provided tailored feedback.	76% of parents called at least once. Of parents who called more than once, the mean number of total calls was 9.1 (5.2).

## Data Availability

Not applicable.

## Supplementary Materials

The following supporting information can be downloaded at: https://www.mdpi.com/article/10.3390/ijerph20010410/s1, Table S1. PRISMA 2020 item checklist.

## Appendix A

**Table A1:** Search Strategy.

	Boolean Operators	Keyword(s)	Limitation
Population		1: Pediatric or child or childhood And 2a: obesity or overweight (database: CINAHL and ‘Academic search premier’) 2b: pediatric or child or childhood or children or pediatrics (database: ‘British nursing index’ and ‘Health premium research’)	‘subject heading’ (Cinahl and Academic search premier) ‘mainsubject’ (British nursing index and health premium research)
Population	and	parent* or caregiver* or mother* or father*	abstract
Exposure	and	telehealth or telemedicine or telecommunication* or ‘digital health’ or eHealth or ‘e-health’ or ‘electronic health’ or ‘m-health’ or mHealth or mobile* or ‘cell phone*’ or cellphone* or ‘mobile application*’ or telephone* or smartphone* or ‘smart phone*’ or ‘text messag*’ or SMS or tablet or app* or ‘e-mail*’ or online or internet or ‘web-based’ or ‘social media’ or web* or computer* or ‘digital tool*’ or ‘digital communication’ or technology or video*	abstract
Outcome	and	evaluation or ‘behavior* change*’ or attitude* or view* or perspective* or management or ‘health education’ or ‘lifestyle change*’ or ‘follow up’ or feasibility or satisfaction or perception* or experience* or opinion* or thoughts	abstract

For additional information, see the reference list.

**Table A2:** Quality Appraisal.

Question Number:	1	2	3	4	5	6	7	8	9	10	11	12	13	Overall
JBI clinical appraisal checklist for randomised controlled trials [49]														
Armstrong et al. (2018) [30]	Y	Y	Y	?	N	?	Y	N	Y	Y	Y	Y	Y	Included
Bala et al. (2019) [31]	Y	Y	Y	Y	N	?	Y	Y	Y	Y	Y	Y	Y	Included
Chai et al. (2021) [32]	Y	Y	Y	?	Y	Y	Y	N	?	Y	Y	Y	?	Included
Davis et al. (2016) [33]	Y	Y	Y	?	N	?	Y	Y	?	Y	Y	Y	?	Included
Johansson et al. (2020) [34]	Y	Y	Y	N	N	?	Y	Y	Y	Y	Y	Y	Y	Included
Sze et al. (2015) [35]	Y	Y	Y	Y	N	N	Y	Y	Y	Y	Y	Y	Y	Included
Taveras et al. (2017) [36]	Y	Y	Y	Y	N	?	Y	Y	Y	Y	Y	Y	Y	Included
Wright et al. (2013) [37]	Y	Y	N	N	N	?	Y	Y	Y	Y	Y	Y	Y	Included
JBI clinical appraisal checklist for qualitative research [48]														
Lee et al. (2019) [38]	?	Y	Y	Y	Y	Y	Y	Y	Y	Y				Included
Parra-Soto et al. (2020) [41] *	?	Y	N	?	?	N	N	N	Y	?				Not included
Sharifi et al. (2013) [39]	?	Y	Y	Y	Y	?	N	Y	Y	Y				Included
Thorén et al. (2021) [40]	?	Y	Y	Y	Y	Y	Y	Y	Y	Y				Included
JBI clinical appraisal checklist for quasi-experimental studies [49]														
Pbert et al. (2016) [44]	Y	N	?	N	Y	N	N	N	Y					Not included
Parra-Soto et al. (2020) [41] *	Y	Y	?	N	Y	Y	-	-	Y					Not included
Militello et al. (2016) [43]	Y	Y	-	N	Y	Y	-	-	Y					Included
Tripicchio et al. (2017) [45]	Y	N	Y	Y	Y	Y	Y	Y	Y					Included
JBI clinical appraisal checklist for cross-sectional studies [47]														
Mulgrew et al. (2011) [42]	Y	N	Y	N	Y	Y	N	Y						Included

Y = Yes; N = No; ? = unclear; - = Not applicable; For some of the studies, we had to go back to the study protocol or other studies referred to in the study in order to make a quality assessment. * = The mixed methods study was appraised using both the qualitative research checklist [48] and the quasi-experimental studies checklist [49].

A keyword summary of experiences from the different studies, divided into themes and by the technological components used. Only findings that included around half or more of the participants are included. For additional information, see the reference list.

**Table A3:** Summary of Experiences.

	Theme 1	Theme 2	Theme 3	Theme 4
SMS	Enjoyed, personalised [30]; Would continue to use/ recommend to others [30,43]; Might continue to use 50/50 [32]; Frequency right [30,32,35,39,43]; Helpful [30,32,39,43]; Relevant, motivating, easy to understand [32]; Timing right [32,39,43]; Satisfied [31,36]; Useful prompts [35,39]	Easy to use, hard to ignore, convenient, some preferred another medium, brief, hard to refer, asynchronous, space limits, wish for an option to change frequency, suggestion multimodal [39]; Initially hard to understand [43]; Easy access to clinicians [34]	Almost 50% worked better with a partner [31,32]; Easy to share [39]; Option to interact [39]; Easy access to clinicians [34]	Personalised [30,39]; Wish for an option to customise [39]; Wanting specific advice, ‘voice of authority’ [39]
Phone	Satisfied [31,32,33,42]; Would continue to use/ recommend to others [31,32,37,42,45]; Saves time and money [31]; Informative, convenient, time appropriate [32]; Helpful [32,33,45]; Useful [32,37]	Mostly no problems [31]; Easy to use [32,37]; Liked to use at home, liked to use on the phone, but 50% would prefer a website [37]	Interaction [31,32]; Clinician approachable [32]; Wanting a face-to-face interaction [33]	Personalised, appropriate [32]; Made for people like them [37]
Web- site	Satisfied [31,36]; Would continue to use [32,40]; Useful, enjoyed [40]; Divided as to usefulness [35]	Easy to use [32,35,40]; Few had problems [32]; Always accessible [40]; Liked one weblink [35]	Request to involve children, family, and friends, wanting community function, liked coaching function [40]	Divergent regarding the amount of information [40]
App	Would continue to use [45]; Helpful [34,45];			
Social media	Effective prompts, timing, and frequency right, a little under half would continue to use [32]; Useful, motivating [38]	Easy to understand [32]; Practical to use at home [38]	Peer support, involvement with a school-based programme [38]	Some felt it was not customised for children [38]
